# A new species of *Dischidia* (Apocynaceae, Asclepiadoideae) from North-eastern Thailand

**DOI:** 10.3897/phytokeys.144.47977

**Published:** 2020-03-17

**Authors:** Wilawan Promprom, Wannachai Chatan

**Affiliations:** 1 Department of Biology, Faculty of Science, Mahasarakham University, Kantharawichai District, Mahasarakham Province, 44150, Thailand Mahasarakham University Mahasarakham Thailand

**Keywords:** Marsdenieae, plant diversity, taxonomy

## Abstract

*Dischidia phuphanensis* Chatan & Promprom, a new species from north-eastern Thailand, is described and illustrated. The new species is similar to *D. tonkinensis* Costantin, but is distinguished by the shape of its leaves, the apices of the staminal corona lobes, the colour of the corolla and the absence of a corolline corona. The distinguishing characters of similar species are discussed. A key for the identification of those species in Thailand without pitcher-like leaves is provided.

## Introduction

*Dischidia*Brown (1810: 461) belongs to the tribe Marsdenieae of the Apocynaceae and comprises approximately eighty species, distributed in India, Indochina, Malesia, Melanesia and the eastern Pacific (Rintz 1980; Livshultz et al. 2005). Some members of *Dischidia* are epiphytes living in association with ants (Livshultz et al. 2005; Kidyoo and Suddee 2017). Ants may inhabit the pitcher-shaped leaves of certain species or they may live in the shelter of those with circular leaves which are convex above and concave below. *Dischidia* exhibits umbel-like inflorescences with small, more or less urceolate flowers that usually have a ring of hairs inside the mouth of the corolla tube and an anchor-shaped staminal corona (Rintz 1980, Forster 1996, Hoffmann et al. 2002). In Thailand, nineteen species of *Dischidia* were reported by Thaithong et al. (2018). During a floristic survey of the forests in north-eastern Thailand conducted between 2009 and 2018, specimens of *Dischidia* were collected in Sakon Nakhon Province. After the living plants and herbarium specimens were carefully investigated, the authors concluded that these could not be referred to any previously named species. Consequently, a new species, *D. phuphanensis*, is described here.

## Material and methods

Specimens were collected from Phu Pha Yol National Park, Sakon Nakhon Province, Thailand in 2017. Morphological observations of the new species were carried out on living plants from the field, as well as on herbarium specimens in BK and BKF. Measurements were made with a Vernier caliper or with an ocular micrometer in a dissecting microscope. We consulted the relevant taxonomic literature (e.g. Kerr (1951), Kidyoo and Suddee (2017), Thaithong et al. (2018) etc). We assessed the preliminary conservation status of the new species using our field knowledge and by applying the criteria given by IUCN (2017).

## Taxonomy

### Dischidia phuphanensis

Taxon classificationPlantaeGentianalesApocynaceae

Chatan & Promprom sp. nov.

74176D46-E5B6-5548-A1CB-55E19C540BD0

urn:lsid:ipni.org:names:77208268-1

Figures 1 , 2

#### Diagnosis.

**Figure 1.**
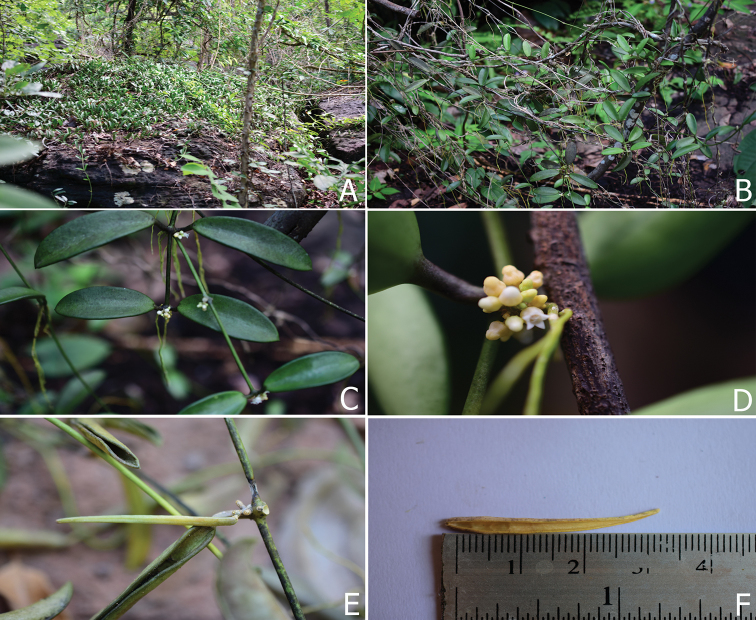
*Dischidia phuphanensis*. **A** Plant climbing on rock **B** plant climbing on branches of shrub 2–3 m tall **C** branches and leaves **D** inflorescence **E** follicle (nearly mature) **F** dehiscent follicle. Photographed by Wannachai Chatan from *W. Chatan 2489* (**A–D**) and *W. Chatan 2904* (**E, F**).

**Figure 2.**
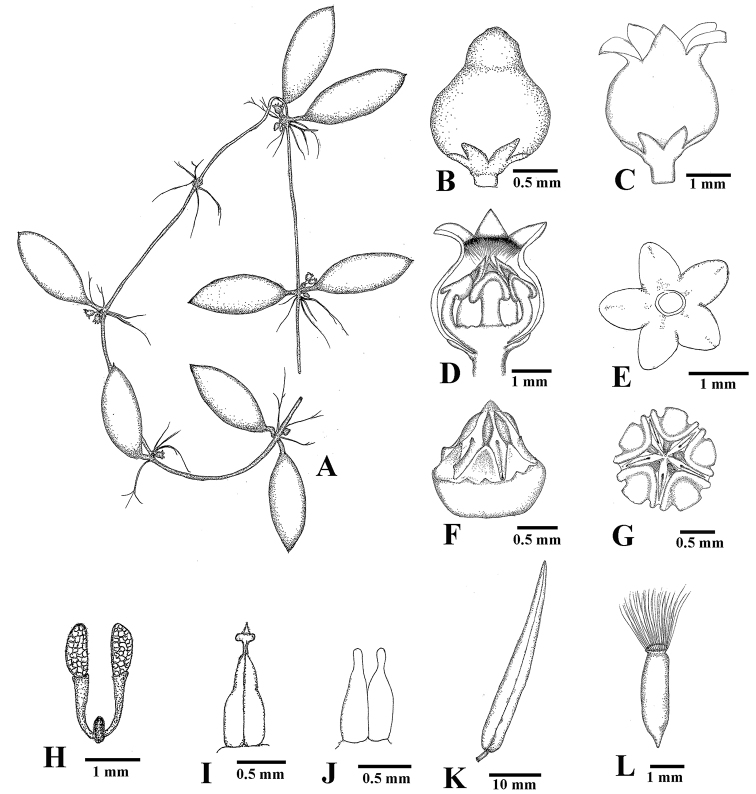
*Dischidia phuphanensis*. **A** Branch with leaves and inflorescences **B** bud **C** flower **D** dissected flower with half of corolla removed **E** calyx from abaxial side **F** gynostegium (side view), with coronal lobes removed **G** gynostegium (from above) **H** pollinarium **I** pistil **J** pistil (style-head removed) **K** follicle **L** seed **A–J** drawn by Wannachai Chatan from *W. Chatan 2489*, and **K, L** from *W. Chatan 2904*.

*Dischidia phuphanensis* is most similar to *D. tonkinensis*, but the new species differs from the latter in its elliptic or narrowly elliptic or slightly oblanceolate leaves (leaves in *D. tonkinensis* are ovate to ovate elliptic, rarely obovate), apex of corona lobes obtuse (with tips pointing downward in *D. tonkinensis*), yellow base of the corolla tube and light yellow or white apices of the lobes (white or orange-yellow corolla tube and lobes in *D. tonkinensis*) and the absence of a corolline corona (corolline corona present in *D. tonkinensis*). (Figures 1, 2)

#### Type.

**Thailand**: Sakon Nakhon Province, Phu Pha Yol National Park, 300–400 m, 16°56.126667'N, 104°2.336667'E, 7 August 2017, *W. Chatan 2489* (holotype: BKF!; isotype: BK!).

#### Description.

***Plant*** epiphytic or lithophytic, sometimes pendulous, fleshy, growing loosely rooted on the host trees or shrubs or on rocks, glabrous except in tube of corolla. ***Branches*** terete, 2.0–2.5 mm thick, green or greenish-purple; internodes 5–15 cm long. Stipular colleters paired, triangular, ca. 0.1 mm long. ***Leaves*** with cylindrical (slightly flattened above), 7–13 × 2.0–2.3 mm petiole; lamina green, dark green or purplish-green, underneath lighter green, slightly fleshy and coriaceous, elliptic, narrowly elliptic or slightly oblanceolate, 3–5 × 1.5–2.0 cm, apex acute-apiculate, base round or slightly acute, margin entire, gland present on adaxial side near lamina base, midrib and secondary veins inconspicuous on both surfaces. ***Inflorescences*** umbelliform, usually bearing 1–4 open flowers and 2–5 developing buds; bracts 2 subtending each flower, triangular, ca. 0.5 × 0.5 mm, greenish-brown, apex acute; peduncle extra-axillary or apparently axillary, persistent, 0–3 mm long; rachis 1–5 per peduncle, bearing scars of previous flowerings 1–3 × 1.0–1.8 mm; pedicels 1–2 × ± 0.5 mm. ***Sepals*** greenish-white, lobes ovate, 0.6–0.8 × 0.5–0.8 mm, apex round, without colleters. ***Corolla*** broadly urceolate or slightly globose, 2.4–2.6 × 2.4–2.5 mm, basally yellow, progressively fading into light yellow or white at the tips of the lobe, corolla tube with one ring of retrorse hairs in throat; lobes triangular to deltate, light yellow or white, 1.3–1.5 × ca. 1.3 mm, apex acute; corolline corona absent. ***Gynostegium*** conical in outline, 1.8–2.0 mm tall, 1.4–1.6 mm in diameter. subsessile; stipe ca. 0.1 mm tall. ***Staminal corona* lobes** anchor-shaped, stalk ca. 0.8 mm high, apical part ca. 0.6 × 0.8 mm, apex obtuse. ***Pollinarium*** erect, ca. 2 mm long. ***Pollinium*** yellow, 0.6–0.8 × 0.20–0.22 mm, ellipsoidal; translator arms 1.0–1.2 mm long; corpusculum ovate, reddish-brown, 0.15–0.16 × 0.05–0.07 mm. ***Ovary*** bicarpellate, bottle-shaped and slightly flattened, 0.7–1.0 mm long, each carpel ca. 0.2–0.5 mm in basal diameter. ***Follicles*** solitary by abortion. linear, 38–45 × 2.5–3.0 mm, green when immature changing to brown when ripe. ***Seed*** slightly cylindrical, 3.8–4.0 × 1.3–1.5 mm, base obconic, bearing white coma 30–32 mm long.

#### Additional specimen examined.

Thailand, Sakon Nakhon Province: Phu Pha Yol National Park, 300–400 m alt., 16°56'07.2"N, 104°02'21.1"E, 5 September 2017, *W. Chatan 2904* (paratype: BKF).

#### Phenology.

Flowering in July–September and fruiting in Aug–December.

#### Distribution.

**Figure 3.**
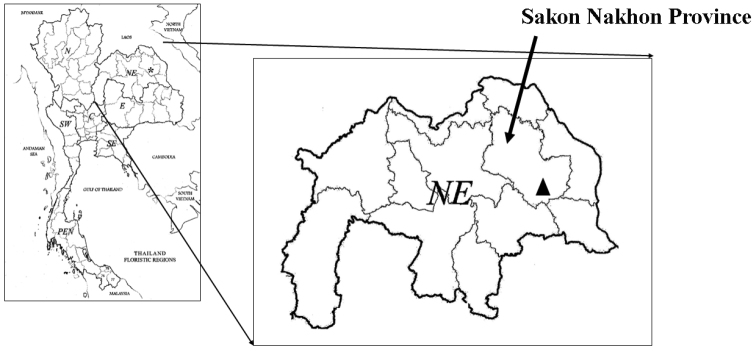
Distribution of *Dischidia phuphanensis* (black triangle) in Phu Pha Yol National Park, Sakon Nakhon Province, Thailand.

The new species is endemic to Thailand and is known only from the type locality, Phu Pha Yol National Park, Sakon Nakhon Province, north-eastern Thailand (Figure 3).

#### Ecology.

This new species grows in both slightly open and in shaded areas in mixed deciduous forest at an elevation of 300–400 m.

#### Vernacular name.

Thao Rag Noi.

#### Etymology.

The specific epithet of *Dischidia phuphanensis* refers to its type locality, the Phuphan mountain range.

#### Preliminary conservation status.

One population of *Dischidia phuphanensis* was found at the type locality in Phu Pha Yol National Park, Sakon Nakhon Province, north-east Thailand. It is estimated to number fewer than 250 mature individuals. Therefore, it should be considered as “Endangered (EN)” according to the IUCN criteria D (IUCN 2017).

#### Discussion.

*Dischidia phuphanensis* is similar to *D. tonkinensis*, from China, Indochina and Thailand (Thaithong et al. 2018). Similarities include their stems (thick, succulent, 2–3 mm in diameter, glabrous), glabrous petiole and lamina, succulent and coriaceous leaves and their glabrous corolla lobes. However, the new species differs from *D. tonkinensis* in its elliptic or narrowly elliptic or slightly oblanceolate leaves, the obtuse apex of the staminal corona lobes, the yellow base of the corolla tube, the light yellow or white apices of the lobes and absence of a corolline corona. *Dischidia tonkinensis* has ovate to ovate-elliptic, or rarely obovate lamina, the apices of the staminal corona lobes are retuse; it has a white or orange-yellow corolla tube and lobes and possesses a corolline corona. The new species is similar to *D. acuminata* Costantin, from Vietnam, in that they share the 1–5 branches to the inflorescence, the short peduncle 0–3 mm long and the absence of a corolline corona. It differs from *D. acuminata* by the triangular to deltate corolla lobes and with acute apices (narrowly shape, thick and abaxial side nose-like in *D. acuminata* (Constantin 1912))

The genus *Dischidia* may be divided into two main groups based on the leaf types, i.e. those with pitcher-like leaves and species with non-pitcher-like leaves. *Dischidia phuphanensis* has non-pitcher-like leaves. The most recent revision of *Dischidia* in Thailand was by Thaithong et al. (2018) and nineteen species were recognised. This was made up of one species with pitcher-like leaves and 18 species with non-pitcher-like leaves. After this new species is added to this group, the number of species with non-pitcher-like leaves is 19. A key to the species with non-pitcher-like leaves in Thailand is provided below and is modified from Thaithong et al. (2018). Details of the morphological differences between *D. phuphanensis* and *D. tonkinensis* are presented in Table 1.

**Table 1.** Distinguishing features between *Dischidia phuphanensis* and *D. tonkinensis*.

Characters	*D. phuphanensis*	*D. tonkinensis*
1. Leaf shape	elliptic or narrowly elliptic or slightly oblanceolate	ovate to ovate-elliptic, rarely obovate
2. Corolla colour outside	tube yellow at base, light yellow or white towards apices of lobes	white or orange-yellow tube and lobes
3. Corolline corona	Absent	present
4. Staminal corona lobes	stalked, anchor shaped with apex obtuse	stalked, anchor shaped with apex retuse

##### Key to species of *Dischidia* with non-pitcher-like leaves in Thailand

**Table:** 

1	Leaves broadly ovate or orbicular or orbicular-peltate; if elliptic, then mixed with others that are peltate or orbicular	**2**
–	Leaves narrowly ovate, elliptic, narrowly elliptic, obovate, lanceolate, oblanceolate, or spathulate; not mixed with peltate or orbicular leaves	**5**
2	Leaves broadly ovate or orbicular or elliptic, abaxial sides slightly flattened	***D. nummularia***
–	Leaves peltate or orbicular, abaxial sides distinctly concave;	**3**
3	Branches pubescent	***D. astephana***
–	Branches glabrous	**4**
4	Staminal corona lobes ± absent	***D. imbricata***
–	Staminal corona lobes consisting of spreading horn-like projections	***D. cornuta***
5	Leaves abruptly laterally expanded at the middle or in upper half	***D. singularis***
–	Leaves not expanded in upper half	**6**
6	Leaves linear or narrowly elliptic or spathulate; proportion of length /width is 3.8–16	***D. bengalensis***
–	Leaves ovate, elliptic, lanceolate, obovate, slightly oblanceolate or broadly obovate; proportion of length/width ratio smaller than 3.8	**7**
7	Leaves broadly obovate, rarely elliptic; staminal corona lobes broadly saddle-shaped	***D. griffithii***
–	Leaves ovate, elliptic, slightly elliptic, lanceolate, slightly oblanceolate; staminal corona lobes anchor shaped or sagittate or reduced to minute swellings	**8**
8	Branches pubescent, tomentose or hirsute	**9**
–	Branches glabrous	**11**
9	Corolla pink, red, dark red or purple, 6–7 mm long, with two rings of hairs inside around mouth of tube	***D. hirsuta***
–	Corolla white or creamy white, less than 5.5 mm long, mouth of corolla tube glabrous or with a single ring of hairs	**10**
10	Corolla ribbed inside; apices of corona lobes sagittate	***D. rimicola***
–	Corolla smooth inside; apices of corona lobes cuneiform	***D. tomentella***
11	Staminal corona lobes reduced to minute swellings	***D. kerrii***
–	Staminal corona lobes stalked and anchor-shaped or sagittate	**12**
12	Branches and leaves succulent	**13**
–	Branches and leaves not succulent	**14**
13	Apices of staminal corona lobes obtuse	***D. phuphanensis***
–	Apices of staminal corona lobes retuse	***D. tonkinensis***
14	Corolla tube and lobes glabrous inside	***D. calva***
–	Corolla with hairs at mouth of tube or on adaxial side of lobes	**15**
15	Corolla with two distinct rings of hairs only in mouth of tube	***D. fruticulosa***
–	Corolla with a single ring of hairs around mouth of tube	**16**
16	Corolla lobes adaxially pubescent	**17**
–	Corolla lobes adaxially glabrous	**19**
17	Corolla lobes triangular, with a ring of hairs around base only	***D. albida***
–	Corolla lobes lanceolate, with hairs from base to the middle	**18**
18	Leaves 1.5–3.0 × 0.7–1.3 cm; corona lobes anchor-shaped with rounded apices	***D. tricholoba***
–	Leaves 2.5–6.5 × 0.9–2.5 cm; corona lobes sagittate with obtuse or truncate apices	***D. singularis***
19	Corolla greenish-white with purple lines alternating with lobes, mouth of corolla tube hairy throughout	***D. punctate***
–	Corolla white or creamy white, with a ring of hairs in mouth of tube	***D. acutifolia***

## Supplementary Material

XML Treatment for Dischidia phuphanensis
